# Investigation of Concurrent Pneumococcal Meningitis in Two Children Attending the Same Day-Care Center

**DOI:** 10.3389/fped.2022.945767

**Published:** 2022-07-14

**Authors:** Alexis Rybak, Emmanuelle Varon, Elodie Masson, Anne Etchevers, Daniel Levy-Brühl, Naïm Ouldali, Corinne Levy, Robert Cohen

**Affiliations:** ^1^ACTIV, Association Clinique et Thérapeutique Infantile du Val-de-Marne, Créteil, France; ^2^AFPA, Association Française de Pédiatrie Ambulatoire, Orléans, France; ^3^Assistance Publique–Hôpitaux de Paris, Clinical Epidemiology Unit, Robert Debré University Hospital, ECEVE INSERM UMR 1123, Université de Paris, Paris, France; ^4^Assistance Publique–Hôpitaux de Paris, Pediatric Emergency Department, Robert Debré University Hospital, Université de Paris, Paris, France; ^5^Université Paris Est, IMRB-GRC GEMINI, Créteil, France; ^6^Laboratory of Medical Biology and National Reference Centre for Pneumococci, Intercommunal Hospital of Créteil, Créteil, France; ^7^Assistance Publique–Hôpitaux de Paris, Pediatric Emergency Department, Bicêtre University Hospital, Paris-Saclay University, Le Kremlin-Bicêtre, France; ^8^The National Public Health institute, Saint-Maurice, France; ^9^Assistance Publique–Hôpitaux de Paris, Department of General Pediatrics, Pediatric Infectious Disease and Internal Medicine, Robert Debré University Hospital, Université de Paris, Paris, France; ^10^Infectious Diseases Division, CHU Sainte Justine - Montreal University, Montreal, QC, Canada; ^11^Clinical Research Center (CRC), Centre Hospitalier Intercommunal de Créteil, Créteil, France; ^12^Neonates Department, Centre Hospitalier Intercommunal de Créteil, Créteil, France

**Keywords:** invasive pneumococcal disease, cluster, pneumococcal carriage, pneumococcal meningitis, daycare center

## Abstract

Only a few clusters of invasive pneumococcal disease have been described globally in children, and most of these cases occurred before pneumococcal vaccination implementation. Two unusual cases of pneumococcal meningitis, occurring in the same daycare center over a 3-day period, were reported. Both cerebrospinal fluid (CSF) were sent to the National reference center for pneumococci. In addition, we decided to perform a pneumococcal carriage study on all children and staff of the daycare center to analyze the pneumococcal serotypes circulating in this DCC and to discuss an antibiotic chemoprophylaxis. CSF culture was positive for pneumococcus, and serotype 25A was identified by latex agglutination. The second case had negative CSF culture, but CSF antigen test and gene amplification results were positive for *Streptococcus pneumoniae*. Serotype 12F was identified by using molecular biology. The absence of correlation between these strains was confirmed by multi-locus sequence typing. In the carriage study, we included 29 children (median age 1.9 years, interquartile range 1.4–2.5) and 10 adults. Among the children, 24 carried *Streptococcus pneumoniae* (83%). The main serotypes isolated were 23A for 6 children and 25A for 5 children; serotypes were non-typeable for 3 children. Only 1 of 10 adults tested carried *Streptococcus pneumoniae* (serotype 12F). Despite this temporo-spatial pattern, the cases were unrelated and not due to carriage of a particular serotype. No specific action has been taken for the other children attending this DCC, and no other case of bacterial meningitis occurred.

## Introduction

*Streptococcus pneumoniae* (Sp) is a major cause of community-acquired invasive bacterial infections worldwide, with more than 300,000 deaths in children aged 1–59 months in 2015 (1). Among invasive pneumococcal disease (IPD), bacterial meningitis has lowest incidence rate and the highest case fatality rate. Pneumococcal disease is always preceded by nasopharyngeal colonization highlighting the importance of carriage studies (2). Carriage is particularly frequent in young children, particularly in children with siblings and/or attending day care center (DCC) (3).

Contrary to *Neisseria meningitidis* or *Haemophilus influenzae*, for which type b was responsible of the majority of cases, only a few clusters of invasive pneumococcal disease have been described globally in children and most of these cases occurred before pneumococcal vaccination implementation (4–7). Furthermore, there is no mandatory declaration of IPD to the health authorities in France and no national guidelines for the management of IPD cluster.

About 100 different pneumococcal serotypes have been described. Vaccines have been designed to protect against a limited number of serotypes among those frequently isolated from IPD. In France, 7-valent pneumococcal conjugate vaccine (PCV7) was recommended to all children aged 2 years and younger since June 2006 and was replaced with PCV13 in 2010 without catch up program, with a coverage >90% (8). A dramatic decrease in pediatric invasive pneumococcal disease, more marked for non-meningitis IPD, has thereafter been reported (8). Since January 2015, a slow increase in pediatric IPD, due to non-vaccine serotypes, has been observed (8).

In this context, the Regional Public Health Agency, ACTIV (Association Clinique Thérapeutique Infantile du Val de Marne), and the National reference centre for pneumococci (NRCP) were notified of 2 cases of pneumococcal meningitis in the same DCC over a 3-day period. In order to analyze the pneumococcal serotypes circulating in this DCC and to discuss an antibiotic chemoprophylaxis, we decided to perform a pneumococcal carriage study. In this study, we report the analysis of both cerebrospinal fluids (CSF) and nasopharyngeal samples performed on all children and staff.

## Methods

### Study Design

We conducted a prospective cross-sectional monocentric study.

### Study Data and Settings

The DCC was located in Paris area, France. It had a maximum capacity of 26 places and welcomed 29 children aged 3 years and younger, some of them on a part-time basis. A nasopharyngeal sample was performed for all children and staff using cotton-tipped wire swabs. Swabs were placed in transport medium and transferred within the same day to the NRCP. Data collected included age, sex and antibiotic consumption within 7 days before swabbing.

### Microbiology

For the CSF samples: on the positive CSF culture, antibiotic susceptibility was determined using minimal inhibitory concentration according to the European Committee on Antimicrobial Susceptibility Testing breakpoints and the serotype was determined using latex agglutination. On other CSF sample, for which the culture was negative, the serotype was determined using amplification of the genes *wzh* and *wzx* (capsular sequence typing). Finally, the two CSF samples were characterized using multi-locus sequence typing (MLST).

For the carriage study: Sp were identified using morphology and standard methods. Latex agglutination with antiserum samples provided by the Statens Serum Institute (Copenhagen, Denmark) was used to serotype the strains as described previously (3).

### Ethics

This study was performed on behalf of the Regional Public Health Agency. Parents and staff members signed a written consent after receiving an information letter.

## Results

### Pneumococcal Meningitis Cases

The first pneumococcal meningitis case was a 21-month-old boy with a positive CSF culture. He was vaccinated with 3 doses of 13-valent pneumococcal conjugate vaccine (PCV13) according to the French immunization schedule (2, 4, and 11 months). He was treated with antibiotics and had a favorable outcome. His CSF culture was positive for Sp and serotype 25A was identified by latex agglutination. This strain was susceptible to penicillin and all the antibiotic tested including macrolides.

The second case was a 6-month-old girl. She was vaccinated with PCV13, but it is not known if she had received 1 or 2 doses. Her CSF culture was negative but both CSF and urine antigen test were positive for Sp. The diagnosis of pneumococcal meningitis was confirmed by gene amplification on the CSF sample. She had a favorable outcome with antibiotics. Serotype 12F was identified using molecular biology.

The absence of correlation between these strains was confirmed by MLST.

### Carriage Study

**Figure 1:**
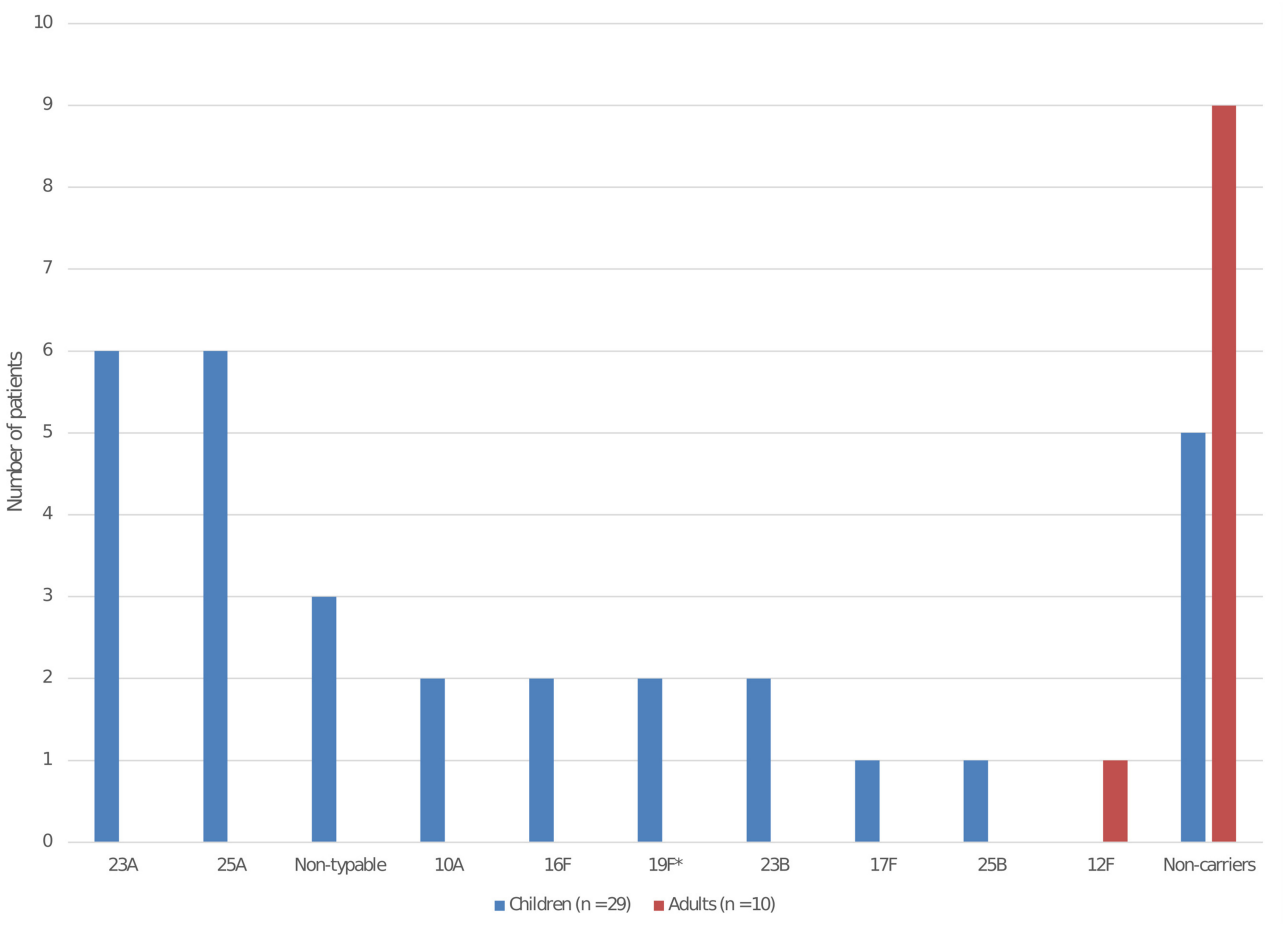
Pneumococcal serotype distribution isolated from nasopharyngeal carriage among the children and adults in the daycare center. *Serotype included in the 13-valent pneumococcal conjugate vaccine. The serotypes involved in the 2 bacterial meningitis were 12F and 25A.

We included 29 children and 10 adults in the carriage study. The median age of children was 1.9 year (interquartile range 1.4–2.5) and 13 were male (45%). Among them, 24 carried Sp (83%). The main serotypes isolated were 23A for 6 children, 25A for 5 children, and non-typable for 3 children (Figure 1). Most serotypes were not included in PCV13 (22/24, 92%). Among the 5 children who did not carry Sp, 2 had recently received antibiotics. Only 1 adult carried Sp among the 10 tested (serotype 12F).

The chronology of the cases and the investigations are in the Table 1.

**Table 1:** Case and investigation chronology.

**Dates**	
17 September 2017	Diagnosis of the first case
19 September 2017	Diagnosis of the second case
20 September 2017	Alert from the Regional Public Health Agency CSF sample from the first case received by the NRCP
22 September 2017	CSF sample from the second case received by the NRCP
25 September 2017	Carriage study performed
28 September 2017	Final report from the NRCP

*CSF, cerebrospinal fluid; NRCP, national reference center for pneumococci*.

## Discussion

In this study, we report 2 cases of pneumococcal meningitis occurring during a 3-day period in children attending the same DCC. Despite this unusual temporo-spatial pattern, the strains were unrelated. Bacterial identification of Sp in sterile sites, defining IPD, and susceptibility patterns are routinely performed by local microbiological laboratories. However, these investigations were not possible for the second case as her CSF culture was negative highlighting the value of molecular investigations which can only be performed by the NRCP. While the absence of relation between these 2 cases was suspected by serotyping, it was confirmed by MSLT which excluded capsular switching. Our study illustrates the need to temporize during the microbiological characterization of the isolated strains despite the call for an immediate reaction. Particularly, an antibiotic chemoprophylaxis to all children has been discussed, with amoxicillin or azithromycin, which is effective to decrease the nasopharyngeal carriage (9), a necessary step for infection (2).

Carriage has been highly modified by PCV implementations. While the overall carriage rate has remained stable, a near disappearance of vaccine serotype with a partial replacement by non-vaccine serotypes has been reported (10). In our carriage study, only 2 children carried a vaccine serotype. Furthermore, we observed a high carriage rate (24/29, 84%) in line with data having shown the high correlation between carriage and DCC attendance (11, 12). Serotype 12F is described as a high invasive disease potential serotype (13) (with low level of carriage and high role in invasive disease) and its involvement in one bacterial meningitis and its near absence in the carriage study was expected: interestingly, serotype 12F was carried by only one adult. By contrast, serotype 25A, responsible of the second case, has not ranked among the serotypes with high invasive disease potential and is usually infrequent in carriage (13). Indeed, between 2012 and 2018, serotype 25A ranked 29th both in carriage and in IPD in France (13).

Multiple reasons may partially explain the occurrence of 2 bacterial meningitis during the same period at the same DCC despite the rarity of this disease. First, the cases were in late September and viral respiratory tract infections are associated with an increased risk of IPD (14). Second, the median age of children with IPD is 1.9 year in France corresponding to children in DCC (15). Third, children attending day care centre are at high risk of both pneumococcal carriage and respiratory tract infections (11, 12). Fourth, a rebound in IPD due to non-vaccine serotypes has been observed in France since January 2015 (8, 10) and both serotypes isolated (12F and 25A) are not included in the PCV13.

The main strength of our study is the responsiveness. Indeed, all our results were available 9 days after the second case occurred. ACTIV and the NRCP have been performing the national surveillance of pneumococcal nasopharyngeal carriage in children in France for twenty years allowing to solve all the logistical problems. This reactivity is critical to have a picture comparable of the time were IPD cases occurred. Carriage is a prerequisite to pneumococcal disease and the risk is particularly high at the beginning of the carriage (16) and is increased when high invasive disease potential serotypes are carried (such as 12F, 24F, 38, 8, 33F, 22F, and 10A) (13). Other factors (age, viral infection, immunodeficiency, vaccination status…) can influence the complex relation between carriage and IPD. Carriage duration of the same serotype is highly variable (17), of about a few days to few months, and might be modified by antibiotic treatment (9), which is frequent in this population (18).

The main limitation of our study is the absence of viral tests. As discussed previously, the cases were in late September and respiratory tract infections could be associated with an increased risk of IPD (14). Viral testing, such as respiratory syncytial virus and influenza testing, could have help us to partially explain the 2 bacterial meningitis. To our knowledge, both children had no medical history. Furthermore, it is not known if the children with bacterial meningitis had immunological investigations to detect an immunodeficiency (19). Since PCV13 implementation, serotypes with lower disease potential were particularly involved in IPD in children with underlying condition (15). However, Gaschignard et al. reported that primary immunodeficiencies were infrequent in children aged <2 years with IPD (3/109, 3%) compared to older children (14/53, 26%; *P* < 0.001). Finally, the 2 children with bacterial meningitis did not participate to the carriage study.

Concurrent cases of IPD are very worrying for parents, DCC staff and physicians taking care of these children, pushing them to prescribe an antibiotic chemoprophylaxis. In France, there are no national recommendations on the management of IPD cluster. In USA and UK, because antimicrobial chemoprophylaxis is unlikely to be beneficial, it is not recommended for contact of children with IPD regardless of their immunization status (20, 21). After the investigations we described, no specific action has been taken for the other children attending this DCC, and no other case of bacterial meningitis occurred. The absence of correlation between the 2 strains involved reinforced the decision to not advise an antibiotic chemoprophylaxis. Our experience and the low level of evidence concerning interventions plaid for the relevance of this strategy.

## Data Availability Statement

The raw data supporting the conclusions of this article will be made available by the authors, without undue reservation.

## Ethics Statement

Ethical review and approval was not required for the study on human participants in accordance with the local legislation and institutional requirements. Written informed consent to participate in this study was provided by the participants' legal guardian/next of kin.

## Author Contributions

EV, CL, and RC designed the study. AR and EM made the acquisition of the study data. AR drafted the initial manuscript and agree to be accountable for all aspects of the work in ensuring that questions related to the accuracy or integrity of any part of the work are appropriately investigated and resolved. All the authors analyzed the data and revised critically the manuscript for important intellectual content, and provide approval for publication of the content.

## Funding

This study was self-funded by ACTIV and by the NRCP.

## Conflict of Interest

Grants for ACTIV were received from GlaxoSmithKline (GSK), MSD, Pfizer, and Sanofi outside the submitted work. AR reports grants from AFPA, and travel grants from Pfizer and AstraZeneca outside the submitted work. NO reports travel grants from Pfizer, Sanofi, and GSK, and grants from the European Society for Paediatric Infectious Diseases outside the submitted work. EV reports grants from French Public Health Agency to the institution, and grants to the institution from Pfizer and MSD, outside the submitted work. CL reports grants to the institution ACTIV from GSK, Sanofi, Pfizer, and Merck, and personal fees and nonfinancial support from Pfizer and Merck, outside the conduct of the study. RC reports grants to the institution ACTIV, personal fees, and nonfinancial support from GSK, Sanofi, Pfizer, and Merck, outside the submitted work. The remaining authors declare that the research was conducted in the absence of any commercial or financial relationships that could be construed as a potential conflict of interest.

## Publisher's Note

All claims expressed in this article are solely those of the authors and do not necessarily represent those of their affiliated organizations, or those of the publisher, the editors and the reviewers. Any product that may be evaluated in this article, or claim that may be made by its manufacturer, is not guaranteed or endorsed by the publisher.
